# Experimental model of type 2 diabetes induced by fat diet consumption and low dose of streptozotocin in C57BL/6J mice

**DOI:** 10.1186/1758-5996-7-S1-A160

**Published:** 2015-11-11

**Authors:** Mariana de Freitas Moreira, Natalia do Vale Canabrava, Sandra Machado Lira, Tatiane Rodrigues Oliveira, José Ytalo Gomes da Silva, Marcelo Oliveira Holanda, Carla Laíne Silva Lima, Julianne do Nascimento Sales, Arnaldo Solheiro Bezerra, Bruno Bezerra da Silva, Erlândia Alves Magalhães Queiroz, Paula Alves Salmito Rodrigues, Raquel Teixeira Terceiro Paim, Lia Magalhães de Almeida, Maria Izabel Florindo Guedes

**Affiliations:** 1 Universidade Estadual do Ceará, Fortaleza, Brazil

## Background

Studies with murine model have been extensively used as a tool in the understanding of mechanisms involved in diseases such as diabetes (Correia-Santos et al, 2012; Chorilli, 2007). Diabetes is characterized as a heterogeneous group of metabolic disorders having in common hyperglycemia, which may result from defects in insulin action or secretion or even in both. The same can be divided into four groups: type 1 diabetes mellitus (DM1), type 2 diabetes mellitus (DM2), gestational diabetes mellitus, and other specific types of diabetes (SBD, 2014). It is known that DM2 is associated with overweight and obesity, physical inactivity, metabolic syndrome, diet high in saturated and animal fat, among others. (Paulweber et al., 2010).

## Objective

To evaluate the effectiveness of induction of type 2 diabetes mellitus Protocol based on a high fat diet associated with low doses of streptozotocin in C57BL/6J mice that simulates the characteristics observed in humans and makes possible subsequent therapeutic propositions and to analyze the consumption of this diet.

## Methodology

83 C57BL/6J mice, with 28 days in the post-weaning period were used. The control animals were divided into experimental groups that received the standard diet for laboratory animals and fat diet composed of a mixture of standard diet, 10% of butter, 1% cholesterol and 0.1% cholic acid for 5 weeks, receiving a low dose of streptozotocin (35mg/kg) at weeks 4 is 5. Both groups (healthy and induced) received the equivalent of 144,3g feed per cage from this point. The animals received food and water ad libitum and such consumption was measured, as well as their weights. Their blood glucose levels were measured on days 0, 35 and 42.

## Results

At the end of five weeks, only 11.68% of induced mice showed corresponding glucose to diabetes. Animals' induction group had a weekly average of 100,41g of feed per cage, making 20,35g of feed/mouse. On the other hand, the control group had an average of feed for cage of 115g, and 19,3g of feed consumption/mouse over this period. As for weight, the healthy group increased on average by 8.11% and induced by 9.03%.

## Conclusion

The animals fed the high fat diet had higher food intake when compared to the control group animals. However, this model for induction of diabetes has proven inefficient.

